# Overcoming chemotherapy drug resistance by targeting inhibitors of apoptosis proteins (IAPs)

**DOI:** 10.1007/s10495-017-1375-1

**Published:** 2017-04-19

**Authors:** Rama Rathore, Jennifer E. McCallum, Elizabeth Varghese, Ana-Maria Florea, Dietrich Büsselberg

**Affiliations:** 1 College of Literature, Sciences and the Arts, University of Michigan-Ann Arbor, Ann Arbor, MI 48109 USA; 2 Weill Cornell Medicine-Qatar, P.O.B. 24144, Doha, Qatar; 3 Institute of Neuropathology, Heinrich Heine University Düsseldorf, Moorenstraße 5, 40225 Düsseldorf, Germany

**Keywords:** Chemotherapy resistance, Inhibitors of apoptosis proteins, Combination therapy, Intrinsic apoptotic pathway, Extrinsic apoptotic pathway

## Abstract

Inhibitors of apoptosis (IAPs) are a family of proteins that play a significant role in the control of programmed cell death (PCD). PCD is essential to maintain healthy cell turnover within tissue but also to fight disease or infection. Uninhibited, IAPs can suppress apoptosis and promote cell cycle progression. Therefore, it is unsurprising that cancer cells demonstrate significantly elevated expression levels of IAPs, resulting in improved cell survival, enhanced tumor growth and subsequent metastasis. Therapies to target IAPs in cancer has garnered substantial scientific interest and as resistance to anti-cancer agents becomes more prevalent, targeting IAPs has become an increasingly attractive strategy to re-sensitize cancer cells to chemotherapies, antibody based-therapies and TRAIL therapy. Antagonism strategies to modulate the actions of XIAP, cIAP1/2 and survivin are the central focus of current research and this review highlights advances within this field with particular emphasis upon the development and specificity of second mitochondria-derived activator of caspase (SMAC) mimetics (synthetic analogs of endogenously expressed inhibitors of IAPs SMAC/DIABLO). While we highlight the potential of SMAC mimetics as effective single agent or combinatory therapies to treat cancer we also discuss the likely clinical implications of resistance to SMAC mimetic therapy, occasionally observed in cancer cell lines.

## Introduction

Cancer develops when cell growth exceeds cell death following a loss in control of the fundamental cellular checkpoints required to maintain healthy tissue turnover. This uninhibited proliferative capacity follows a dysregulation in oncogenic expression that results in tumor formation. In healthy cells, many of these processes give rise to stimuli that promote the induction of apoptosis, most prominently regulated by the B cell lymphoma 2 (Bcl-2) family of proteins [1]. However, in cancer pro-apoptotic factors are suppressed and anti-apoptotic proteins, such as the inhibitors of apoptosis proteins (IAPs) are upregulated, promoting uncontrolled cell division [2]. This excessive rate of cell proliferation gives rise to a hypoxic microenvironment and a dysregulation in growth factors, such as vascular endothelial growth factor (VEGF), that promote angiogenesis and genetic adaptations that can permit a tumor to thrive [3].

In cancer treatment, this dysregulation is targeted via multi-therapeutic approaches that include antibody-based, chemo- and radio-therapy. Most recent data from clinical trials suggest that both chemotherapy and radiation remain best first line therapies for aggressive lung cancer [4], reducing tumor size via stress induced apoptosis following direct and irreparable physical or chemical damage to DNA [5]. Whilst these approaches can be effective in the short term, the maximal dosages required to maintain anticancer agent or radiation effectiveness can, over time, give rise to cancer cells that exhibit chemo- and radio-resistance. Evidence suggests that some high dosage chemotherapy leads to caspase-independent necroptotic cell death, but it remains unclear if toxicity to healthy cells may be a compromising factor in its effectiveness [6]. Some cancer cell types exhibit intrinsic resistance to chemotherapy drugs, often attributed to high endogenous expression of drug efflux transporters such as MDR1 [7] and therapies targeting efflux systems are now in their third generation of development [8]. To combat both intrinsic and acquired chemoresistance, and thus prevent the eventual invincibility of cancer cells, it is important to better understand the role that caspase-mediated apoptosis plays in cancer agent mediated cell death pathways and chemoresistance.

In line with this, the expression and function of anti-apoptotic and pro-apoptotic proteins have long been considered as potential strategies to target cancer pathogenesis via inhibitors and activators, respectively [9]. Already in combinatory cancer treatment, data from clinical studies suggest that classical chemotherapeutic drugs such as paclitaxel exert a synergetic action with pro-apoptotic agents like bortezomib to improve patient survival in radio-resistant non-small cell lung cancer [10]. In the same regard, it has been proposed that targeting IAPs could be equally helpful in combinatory therapy against cancer. Furthermore, modulation of their expression can facilitate direct targeting of the cell’s apoptotic machinery to improve cell death [11]. In relation to chemo-sensitization, IAP modulation is particularly attractive because it bypasses upstream signaling pathways that may be impaired by resistance focusing on target initiator and effector caspases.

This review focuses on the role of IAPs in drug resistance and how to overcome it. To address this, the merits of mono-therapy with IAP-antagonists and combinatorial treatments with chemotherapeutic agents will be discussed. Within a wider perspective, the role of other small molecular inhibitors used in cancer treatment and their potential for co-treatment to target IAPs will be explored. Furthermore, given that some cancer cell types exhibit intrinsic resistance, it will explore the consequences of acquired resistance to IAP-antagonists and small molecular inhibitors in cancer treatment.

The central questions of this review are:

1. How best to target IAPs to overcome drug resistance?
2. How to tackle acquired resistance to IAP antagonism?

These are important questions in the field of cancer treatment and their answers will help to develop more efficient therapies for patients with acquired and intrinsic chemoresistance. Moreover, enhanced therapeutic approaches may improve patient survival in previously difficult to treat or aggressive cancers.

## Apoptosis pathways and cancer

Cancer cells are more resistant to apoptotic cell death, allowing them to bypass critical biological checkpoints that normally maintain cell turnover in healthy tissues. Specifically, checkpoints can fail following an introduction of mutations in apoptotic genes such as p53, or DNA-repair genes like Brac1/2 [12]. Given this, it is unsurprising that the administration of high dose anti-cancer therapies required to kill defective cells can indirectly induce apoptotic cell death in ‘vulnerable’ healthy tissues, producing unwanted side effects. For example, the effect of platinum-based chemotherapy on gastrointestinal tissue health is of particular concern and can be a major hindrance to the success of therapeutic regimens in the clinic [13]. Taking this together, it is suggested that direct manipulation of apoptotic pathways via IAP antagonism can offer a safer alternative that has limited effect on apoptosis in non-cancer cells that do not highly express IAPs [14].

**Fig. 1:**
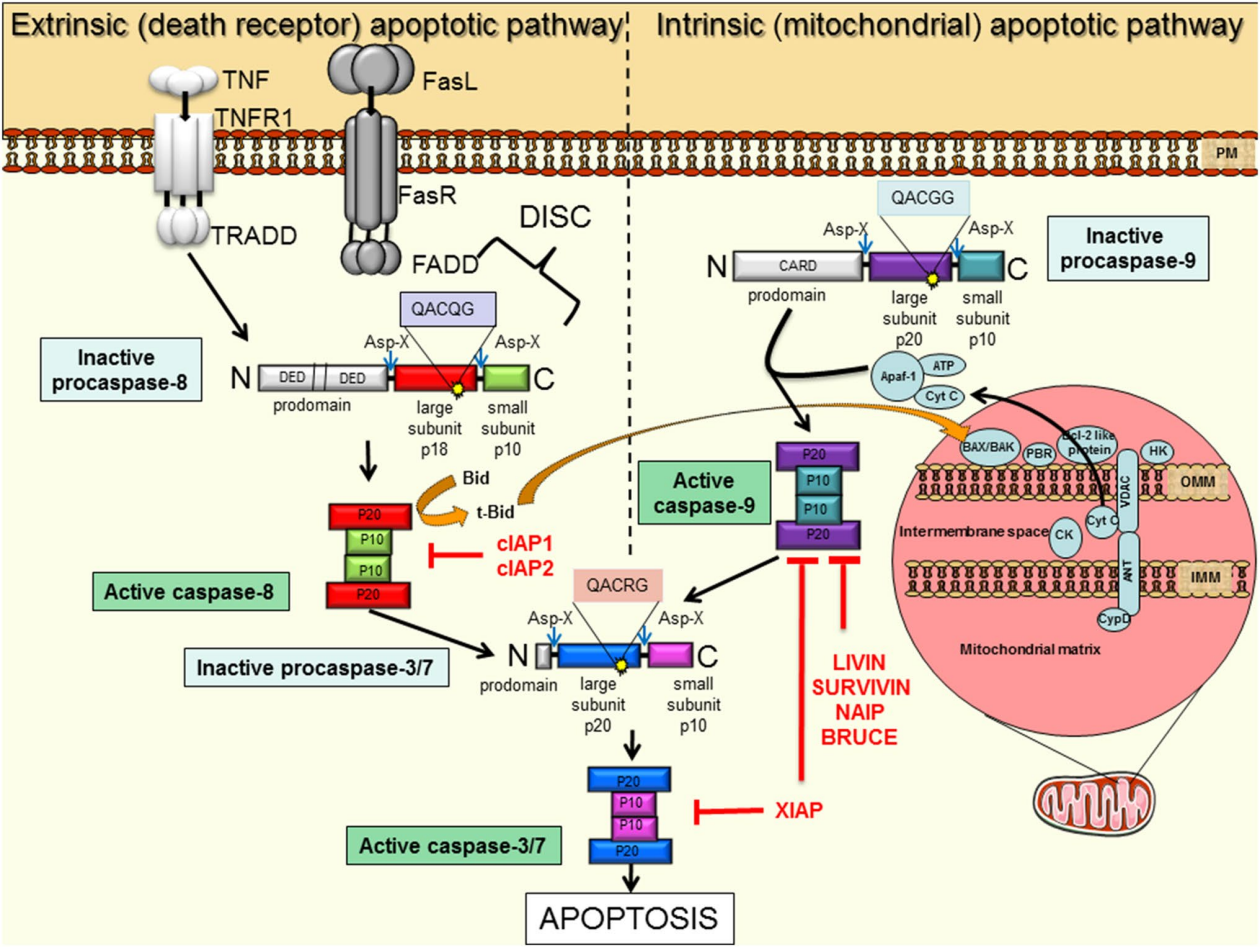
Schematic illustration of the extrinsic and intrinsic apoptotic pathways, as well as the inhibitory effect of various IAPs on pro-apoptotic molecules. Extrinsic apoptotic pathway initiated by binding of death ligands, such as FasL or tumor necrosis factor (TNF) to death receptors located on the plasma membrane. This reaction is followed by the recruitment and binding of molecules like Fas-associated death domain protein (FADD) or tumor necrosis factor receptor type 1-associated death domain protein (TRADD) to the cytosolic domain of death receptors. Death-inducing signaling complex (DISC) is formed by death receptor, FADD and caspase 8. DISC formation initiates the signal transduction that culminates in apoptosis via caspase 3/7 activation. Active caspases can enhance apoptosis via cleavage of Bid to tBid; a cross-talk facilitator that mediates the mitochondrial amplification loop. The truncated Bid (t-Bid) promotes the release of cytochrome c, via Bax, in mitochondria. The intrinsic pathway, is initiated within at the outer mitochondrial membrane (OMM) in response to cellular stress. As a result, these mediate mitochondrial permeability via interaction ‘pro-apoptotic’ Bcl-2 proteins to stimulate release of cytochrome c and SMAC, which bind and inhibit IAPs. Cytochrome c, Apaf-1 and ATP binds to pro caspase 9 leading to apoptosome formation and activation of caspase 9, which in turn activate caspase 3 permitting the cell to proceed to apoptosis. IAPs are endogenous inhibitors of apoptosis identified in humans. The family members XIAP, cIAP1, cIAP2, NAIP, Livin and Survivin and BRUCE can bind caspases to block apoptosis. Importantly, their dysregulated expression is associated with cancer and chemoresistance

There are two major ways in which caspase-dependent apoptosis can be induced; the first is via an activation of the intrinsic apoptotic pathway (also known as the mitochondrial apoptotic pathway) and the second involves activation of the extrinsic pathway (also known as the death receptor or transmembrane apoptotic pathway) [15] (Fig. 1). Though induced differently, cross-talk can facilitate amplification of the extrinsic pathway via the intrinsic pathway, known as the mitochondrial amplification loop [16]. Crucially, both pathways converge at the effector caspase level. Initiator caspases involved in the extrinsic pathway are caspase 8 and caspase 10, while the caspases involved in the intrinsic pathway are caspase 9 and 2. Caspase 3 and 7 are terminal effectors that execute apoptosis in response to stimuli from both intrinsic and extrinsic pathways. Evidence suggests the role of effector caspases are extremely wide ranging and their protease activity has been demonstrated in >400 distinct substrates. Examples include reduction of cellular adhesion proteins such as α-adducin and β-catenin to initiate cell detachment, to subsequent and systematic ‘cell demolition’ via targeting of scaffolding proteins like ROCK and chemotactic factor release that encourages the infiltration of phagocytic cells [17, 18].

### The extrinsic apoptotic pathway

The extrinsic pathway is activated by the binding of ‘death’ ligands to transmembrane receptors. The most prominent examples of these include the trimeric Fas ligand (FasL) and tumor necrosis factor (TNF), which bind to the Fas receptor (CD95/apoptosis-1R) and the tumor necrosis factor receptor (TNFR), respectively (Fig. 1). During receptor activation, the cytoplasmic domain of the ligand bound receptor complex associates with the death domain (FADD) of an adaptor molecule (Fas-associated protein) to enable binding to caspase 8. This facilitates oligomerization and association of death domains on the adaptor molecules with those on the zymogenic pro-caspase 8. Together they form the death-inducing signaling complex (DISC) [19]. DISC mediates autocatalysis and thus, activation of pro-caspase 8 to caspase 8 which initiates the signaling cascade resulting in auto-activation of terminal effector caspases 3 or 7, responsible for the definitive apoptosis signal.

A similar pathway is activated when TNF binds to TNFR, instead promoting association of the TNFR cytoplasmic domain with adaptor molecule TRADD (TNFRSF1A associated via death domain) and pro-caspase 8 [19]. Other intracellular signaling proteins associated with this pathway can either be pro- or anti-apoptotic in nature. For example, the Flice-associated huge protein (FLASH) and the small accelerator of death signaling (SADS) are understood to aid recruitment of pro-caspase 8 to the DISC complex, promoting apoptosis [20]. On the other hand, dysregulation of expression of the protein Bid, responsible for cross-talk between the extrinsic and intrinsic apoptotic pathway (activating the mitochondrial amplification loop), has been shown to potentiate apoptotic survival in hepatocytes [21].

### The intrinsic apoptotic pathway

The intrinsic apoptotic pathway is controlled by the Bcl-2 superfamily that initiate the release of pro-apoptotic proteins from the mitochondrial intra-membrane space [22] (Fig. 1). This includes the efflux of cytochrome c (Cyto c), second mitochondria-derived activator of caspase/direct inhibitor of apoptosis-binding protein with a low isoelectric point (SMAC/DIABLO) and high temperature requirement protein A2 (Omi/HtrA2). Bax proteins, belonging to the Bcl-2 superfamily (discussed further, Fig. 1.) are understood to mediate the opening mitochondrial permeability transition pores (PTPs), disrupting the mitochondrial transmembrane potential and permeability [22]. Often found to be upregulated in cancer and chemoresistance [23], anti-apoptotic Bcl-2 proteins such as Bcl-2, Bcl-XL and Mcl-1 inhibit Bax activation [22].

Currently, the exact mechanism of Cyto c release from the mitochondrial membrane space into the cytosol is not completely understood. Specifically, it is not clear if Cyto c release precedes or follows the opening of PTPs. While the general consensus suggests that Bcl-2 proteins facilitate pore opening [24], some research suggests that Cyto c play a role in maintaining the mitochondrial membrane potential and drives ATP synthesis following an opening of the pores [25]. There are three major types of Bcl-2 protein families; ‘the Bcl-2 anti-apoptotic proteins’, ‘the multi-domain pro-apoptotic proteins’ and the ‘BH3-only pro-apoptotic proteins’. The most prominent, Bax and Bak, belong to the ‘multi-domain’ family and their pro-apoptotic effect can be augmented by members of the ‘BH3-only’ family. BH3-only proteins such as Bim, Bmf, Bad, Bid and Noxa also activate apoptotic machinery by neutralizing the anti-apoptotic Bcl-2 proteins [26].

As previously noted, Bax proteins permeabilize the outer mitochondrial membrane and it is thought that this occurs following translocation of monomeric Bax from the cytosol to the mitochondria [27]. It is still unclear what promotes this translocation, but evidence suggests that alterations in pH may be of importance [28]. Evidence also suggests that Bax undergoes a conformational change, oligomerizes with Bak and undergoes insertion into the outer mitochondrial membrane via it carboxyl terminus [29]. This is a rapid association mediated by a pro-apoptotic Bcl-2 protein called tBid, which can sometimes be inhibited when tBid is bound to Bcl-XL [30]. Contrastingly, Bax can be anchored to voltage-dependent anion channel 2 (VDAC2) on the outer mitochondrial membrane, preventing pore opening [31]. Most recent evidence suggests that VDAC may provide the molecular platform for Bax retrotranslocation to the cytosol following the initiation of pro-survival pathways [32].

Cyto c release into the cytosol is facilitated by its binding to dATP and apoptosis protease activating factor 1 (Apaf1) to form a multimeric complex known as an apoptosome, which has a function similar to that of DISC in the extrinsic pathway (Fig. 1). This apoptosome recruits pro-caspase 9 by interacting with its caspase-recruitment domain (CARD) to cause autocatalysis and activation of caspase 9, initiating the caspase signaling cascade. Eventually, effector caspase 3 is activated and apoptosis is induced [22]. The intrinsic apoptotic pathway also initiates caspase-independent apoptosis. Mitochondrial proteins such as Omi/HtrA2 and the apoptosis inducing factor (AIF) are able to initiate caspase-independent apoptosis via programmed cell death (PCD) [33]. Whilst caspase-independent mechanisms of PCD remain least well understood, in vitro evidence suggests that targeting these proteins and other caspase-independent signaling like RIPK-3 mediated necroptosis could potentiate cancer cell death and thus supersede the requirement to modulate caspase activity, at least via the intrinsic pathway [33, 34]. However, the mechanisms governing the initiation of necroptosis mediated cell death are so far unexplored in vivo and the repercussions of its potential pro-inflammatory nature have not been fully elucidated [34, 35].

The extrinsic and intrinsic apoptotic pathways induced in response to chemotherapy or radiotherapy indirectly induce caspase-dependent apoptosis in cancer cells. Terminal caspases 3 and 7 activate nucleases, cytoplasmic substrates and multiple degradation enzymes to trigger PCD [36]. Importantly, IAPs prohibit the activation of caspase 3 and 7 following activation of extrinsic and intrinsic pathways of apoptosis, alike. Targeting molecules such as IAPs will relieve the inhibitory stress on caspases and encourage unhealthy chemotherapy-resistant cells to undergo cell death via apoptosis.

## Inhibitors of apoptosis (IAPs)

The execution of extrinsic (death receptor) and intrinsic (mitochondrial) apoptotic signals are modulated by a family of structurally distinct IAPs; X-linked (XIAP), cellular (cIAP1, cIAP2), neuronal (NIAP), testis specific (Ts-IAP), Bir-ubiquitin conjugating enzyme (BRUCE), Survivin and Livin. Structurally, IAPs are approximately 70 amino acids long and contain zinc finger Baculovirus IAP Repeat (BIR) domains that are responsible for the inhibitory properties of IAPs as they prevent the conversion of zymogenic pro-caspases to active caspases [37]. Whilst IAPs are expressed basally, their expression is preferentially upregulated in both disease and drug resistance. For example, significantly higher expression of all IAP family members was reported in a subset of CD133+ glioblastoma stem cells exhibiting resistance to temozolomide, carboplatin and paclitaxel [38]. Notably, the expression of XIAP and cIAP1 was 21.9 and 39.0-fold higher in resistant compared to sensitive cells, respectively [38]. The characteristics of each IAP member, inclusive of alternative names, structure and expression profiles are outlined in Table 1.

**Table 1:** Inhibitors of apoptosis (IAP) family; alternative nomenclature, structural feature, expression and functional role

lAP	Other names	Features	Expression	Function
NAIP (neuronal apoptosis inhibitory protein)	BIRC 1	3 BIR domains	Central nervous system (CNS), macrophages [39]	Resistance to *Legionella pneuonophilia* infection [40]
		1 NOD domain		Caspase-9 inhibition [41]
		1 LRR domain		Innate immunity and neuroprotection [42]
XlAP (X-Iinked inhibitor of apoptosis)	MIHA	RING domain with E3 ubiquitin ligase activity	Expressed in nucleus and cytoplasm of all mammalian cells [43]	Innate immunity [44]
	hILP	3 BIR domains	Melanoma [45]	Mitotic cell death [46]
	BRIC 4	1 UBA domain	Acute myeloid leukemia (childhood) [47]	Copper homoeostasis [48]
	AP13	Only IAP to directly inhibit caspase actvity	Diffuse large B cell lymphoma [49]	Cancer cell motility [50]
		Found on the X chromosome	Renal cell carcinoma [51]	Can activate NFkB [52]
			Breast cancer [53]	
	LIP-1		X-linked lymphoproliferative disorder (XLP) [54]	
cIAP1 (cellular IAP1)	HIAP 2	3 BIR domains	Esophagus, tonsil, testis, thyroid, skin, lung, and brain [43]	E3 ubiquitin protein ligase activity controls the canonical and non canonical pathways of NFkB signaling [55]
			Multiple myeloma [56]	
			Colo rectal cancer [56]	
			Renal-cell carcinomas [57]	
	MIHB	1 RING domain with E3 ubiquitin ligase activity	Squamous cell carcinoma [58]	Modulates cell cycle [59]
				Involved in MAPK signaling [60]
	BIRC 2	1 CARD domain	Non small cell lung carcinomas [61]	Regulator of innate immune signaling [62]
				Protects cells from spontaneous formation of the ripoptosome [63]
				Regulators of tumor necrosis factor-mediated signaling pathway [55]
cIAP2 (cellular IAP2)	HIAP1	3 BIR domains		
	MIHC	1 CARD domain	Normally seen in fetal lung, and kidney. Esophagus, tonsil, testis, thyroid, skin, lung, and brain etc. [43]	
	API2		Colo rectal cancer [56]	Regulation of tumor necrosis factor-mediated signaling pathway [55]
			Multiple myeloma [64]	Regulator of innate immune signaling [62]
			Breast carcinoma [65]	
			Pancreatic carcinoma [66]	Involved in MAPK signaling [60]
	BIRC 3	RING domain with E3 ubiquitin ligase activity	Bladder cancer [67]	
			Chronic lymphocytic leukemia [68]	
Ts-IAP (testis-specific IAP)	hlLP2	1 BIR domain	Found in normal and cancerous testis	
	BIRC 8	1 RING domain		Restricted specificity for caspase 9 [69]
	ILP-2			Suppess apoptosis induced by Bax but not Fas-mediated apoptosis [69]
BRUCE (BIR-containing ubiquitin conjugating enzyme)	Apollon		High levels in brain,testis and lymphatic cells [70]	Suppression of apoptosis by facilitating the degradation of SMAC and caspase-9 [71] and promotion of cytokinesis [72]
		1 BIR domain		
	BIRC6	1 UBA doma n	Colorectal cancer [73], neuroblastoma, melanoma [74], prostrate cancer [75]	DNA damage repair and genome stability [76]
Survivin	TIAP (murine homologue of human survivin)		Some of the normal adult human tissues, including colonic mucosa, placenta, bone marrow and keratinocytes of the basal layer of the skin. In embryonic tissues [77]	Regulation of physiological functions of haematopoietic stem cells, neuronal stem cells or intestinal stem cells [78, 79]
				Inhibits both Bax, Fas, caspase induced apoptotic pathways [80]
				Regulation of cell proliferation and cell death [81]
	BIRC 5	1 BIR domain	Over-expressed in lung, liver, heart gastrointestinal tract, colon, pancreas, prostrate and breast cancer [82]	Promotes angiogenesis, metastasis and chemo resistance [83–85]
			Found in hematological malignancies, lymphomas, acute leukemias and myelodysplastic syndrome [82]	
Livin	KIAP	1 BIR domain	Normally present in placenta and embryonic tissue, melanoma [86], neuroblastoma [87], lung cancer [88], Gl cancer [89], esophageal [90], osteosarcoma [91]	Specialized function of embryonic development [86]
	ML-IAP	1 RING domain		
	BIRC 7	Has 2 isoforms, Livin-alpha and Livin-beta		

Therapeutic modulation of cells overexpressing IAPs can be approached in multiple ways; by down-regulating their expression to potentiate cell death when apoptotic stimuli are present, or via an up-regulation of natural pro-apoptotic proteins such as Bax, TNF-α or FasL, or by direct inhibition of IAPs action on caspases [92]. Therapeutics that directly antagonize IAPs, or over-express, mimic and increase the potency of pro-apoptotic proteins have been the most widely accessible strategies to date. Dependent on their efficacy, these approaches could work mono-therapeutically or to re-sensitize cancer cells to chemotherapeutic agents by synergizing with them with combinatorial therapies (discussed later, see Table 2).

**Table 2:** A list of prominent SMAC mimetics, their indications for therapeutic strategies in specific cancers, suggested combinatorial approaches and clinical trials conducted to date [39–91, 93–108]

SMAC mimetic	Target	Cancers in vitro	Potential combinatory therapies	Clinical trials
AT-406	XIAP, cIAP1, cIAP2 [93]	Ovarian cancer [109], solid tumors (including breast, head and neck) [94], colorectal cancer [35]	Carboplatin, cisplatin and paclitaxel [109], radiation therapy [96], TRAIL, Bcl-2 and BRAF inhibitors [95]	Phase II, single agent, ovarian cancer (Debiopharm), Phase I acute myelogenous leukemia (AML) (terminated), Phase I (completed in 2014), Dose escalation study (Debiopharm), Phase l/lb non-small cell lung cancer (2016, Debiopharm, Merk and Phizer)
LCL-161	Degradation of cIAP1 and cIAP2 [97, 98]	Multiple myelofibrosis, solid tumors (including breast and ovarian) [99], esophageal squamous cell carcinoma [97], non-small cell lung cancer [98]	TNF- α/TRAIL [99], radiation therapy [97], paclitaxel [110, 98]	Phase II, LCL161 adjunct to cyclophosphamide for multiple myeloma (Ongoing-Mayo Clinic), Phase II, single agent, multiple myelofibrosis (2016, ongoing M.D. Anderson Cancer Center and Novartis), Phase II LCL161 adjunct to paclitaxel for triple-negative breast cancer (completed in 2014, Novartis Pharmaceuticals), Phase I LCL161 adjuct to PDR001 for colorectal, triple-negative breast cancer and (2016, ongoing Novartis Pharmaceuticals)
GDC-0152	XIAP, cIAP1, cIAP2 and ML-lAP [100, 101]	Breast cancer [100], glioblastoma [101]	No information	Phase 1 solid cancers (completed in 2010, Genetech)
BIRINAPANT	Degradation of cIAP1 and cIAP2 [102]	Breast cancer [103], ovarian cancer [104], solid tumours [105], melanoma [106]	Carboplatin [104], TRAIL [102, 103], TNF-α [106]	Phase II ovarian cancer, single agent (completed 2015, National Cancer Institute), Phase I lymphoma, dose escalation study (completed 2013, TetraLogic Pharmaceuticals), Phase Ib, ovarian and peritoneal cancer, Birinapant adjunct to conatumumab (Completed 2015, TatraLogic Pharmaceuticals), Phase Ib/2a, myelodysplastic syndrome, birinapant adjuct to 5-azacitidine (completed 2015, TetraLogic Pharmaceuticals), Phase II, advanced or recurrent high grade carcinoma, birinipant adjuct to platinum based agents (initiated 2017, Jonsson Comprehensive Cancer Center and TetraLogic Pharmaceuticals). Phase I/II, solid tumors, dose escalation study in combination with pembrolizumab (initiated 2017, TetraLogic Pharmaceuticals)
HGS-1029	XIAP inhibition, loss of cIAP expression [111]	Advanced solid tumors (including colon, andrenocarcinoma) (TetraLogic Pharmaceuticals)	No information	Phase I, single agent for solid tumors (completed in 2012, Aegera Therapeutics)
BV6	XIAP, cIAP1 and cIAP2 [107, 108]	Breast cancer [112], acute myeloid leukemia (AML) [107], childhood ALL [108]	Drozitumab [112], 5-azacytidine [107], dexamethasone [108]	N/A

### Modes of direct IAP antagonism

Studies assessing knockout strategies in cancer cells with high endogenous expression of IAPs have been essential in highlighting their role in the maintenance of resistance to various anti-cancer therapies. For example, shRNA mediated knockdown of XIAP re-sensitized ovarian cancer cells to cisplatin therapy and suppressed tumorigenicity in nude mice via increased apoptosis [113]. Similar findings of reduced tumorigenicity, reduced angiogenesis and improved apoptosis were reported following shRNA mediated knockdown of Survivin in breast and ovarian carcinoma in vivo [114]. In the clinic, phase II trials initially reported successful outcome in acute myeloid leukemia (AML) patients undergoing therapy using antisense oligonucleotide AEG35156 that target XIAP [115, 116]. Despite this initial success and confirmed on-target knockdown [116], a later trial failed to report a similarly improved outcome in patients with advanced pancreatic cancer [117]. Whilst gene silencing is attractive prospect, its potential clinical relevance is limited by lower knockdown efficiency in patient samples, compared to those demonstrated in cell culture [115] and by the transient nature of XIAP repression [117]. Still, strategies for RNAi remain important tools to dissect the mechanistic and functional role of IAPs in cancer.

**Fig. 2:**
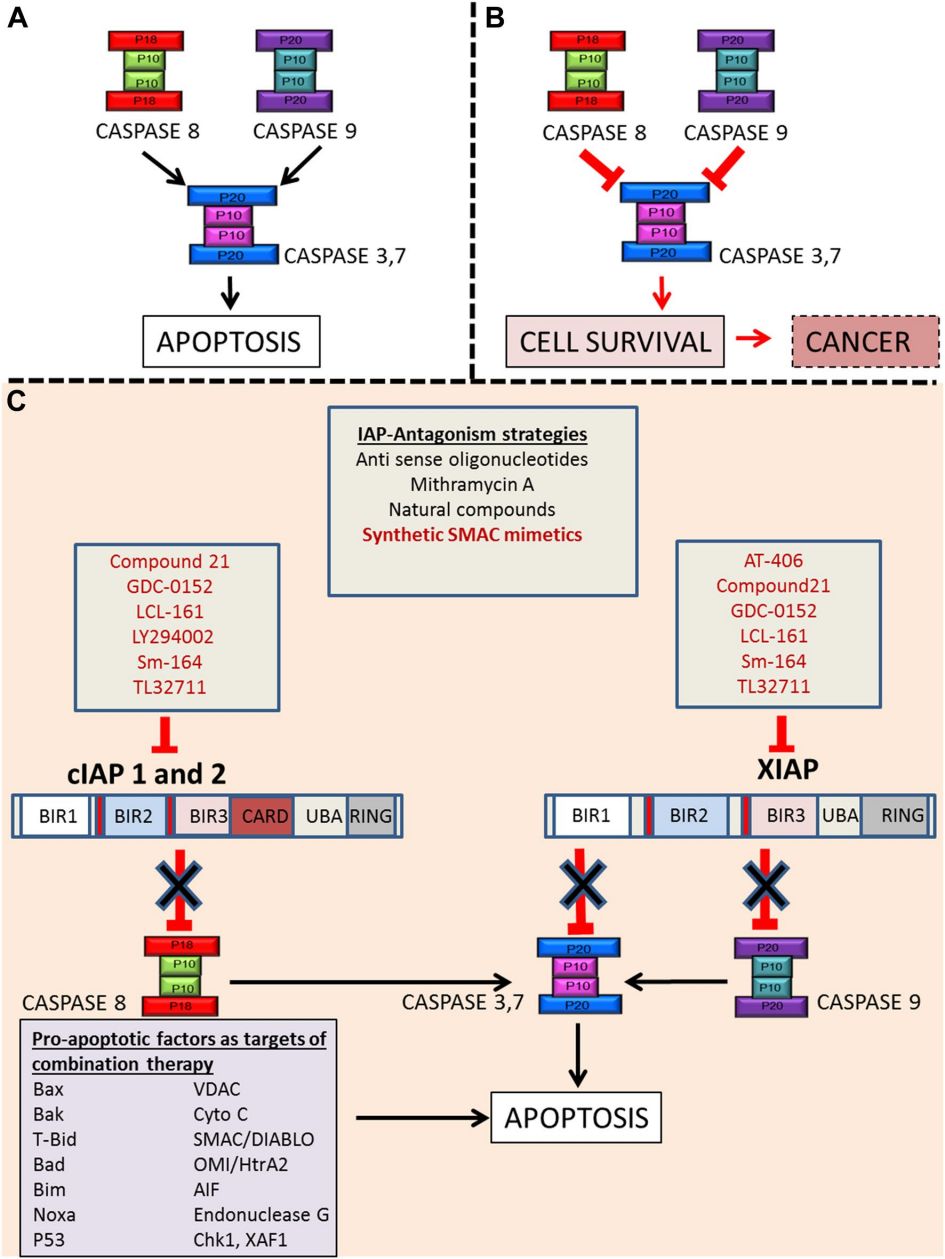
Downstream apoptotic pathways decide cell fate. In physiological conditions, IAPs mediate cell and tissue homeostasis by mediating apoptosis. **a** In normal conditions, caspases are uninhibited and the cell under goes apoptosis and **b** In cancer pathology, the cell escapes apoptosis and proceeds to tumor formation. IAPs are endogenous proteins that inactivate caspases via direct binding, preventing apoptosis thus contributing to oncogenesis and resistance to therapy. **c** Strategies to target IAPs for anti-cancer therapy include RNA knockdown, small molecule inhibitors and SMAC mimetics. SMAC mimetics are listed by their affinity for either XIAP or cIAP1/2 (RED). Also highlighted are various pro-apoptotic factors, often used as parameters, and targets, of successful combinatory therapies that promote apoptosis

Primary IAP antagonists are SMAC mimetics. These are synthetic mimics of an endogenous second mitochondria-derived activator of caspase/direct IAP binding protein with low pI (SMAC/DIABLO) protein, a natural inhibitor of IAPs [118, 119]. Endogenous SMAC/DIABLO exerts its inhibitory effect on IAPs by binding to the zinc-binding baculovirus IAP repeat (BIR) domain of X-chromosome-linked IAP (XIAP) [120], competitively inhibiting its binding with effector caspases-9, 3 and 7, thus preventing their inhibition. Therefore, active caspases remain active and PCD can occur. SMAC binding to IAPs is facilitated by the interaction of its 4 N-terminal residues (alanine–valine–phenylalanine–isoleucine) with BIR domains on XIAP [120] (Fig. 2).

SMAC/DIABLO can also bind to BIR domains of cellular IAPs (cIAPs), promoting their ubiquitination and proteasomal degradation. In turn, this can stimulate the production of TNF-alpha which sensitizes the cell to TNF-alpha dependent apoptosis, further promoting cell death [121]. Although SMAC/DIABLO targets both cIAP and XIAP, it has a greater affinity for the BIR3 domain of XIAP than for the BIR2 domain of cIAPs [122]. Therefore, antagonist action of SMAC mimetics may be optimal in targeting the BIR3 domain of XIAP. However, it is still unclear if single agent therapy is more effective than combinatorial therapy. Figure 2 schematically outlines the structural characteristics of two highly expressed and prominently targeted IAP proteins in cancer; XIAP and cIAP1/2. It also highlights various modes of therapeutic intervention explored to modulate their actions.

Survivin and BRUCE are other prominent IAPs. Outlined in Table 1, these are structurally unique owing to only one BIR domain and therefore, are less easily targeted via SMAC mimetics. Survivin and BRUCE are mechanistically different to other IAPs in that they regulate cytokinesis and multiple mitochondria-mediated signaling pathways, rather than apoptosis (reviewed in [92]). It has been reported that survivin can form a complex with XIAP to improve its stability as an apoptosis suppressor [123] and metastasis promotor [124]. However, it was shown that XIAP antagonist XAF1 can displace survivin to improve tumor cell death [125]. High survivin expression has been reported as the cell’s “Achilles heel” in chemoresistance and it has been suggested as a prominent gene to target anti-cancer therapy resistance in neuroblastoma [126, 127]. shRNA mediated knockdown of survivin is an effective strategy to re-sensitize H292 lung cancer cells to cisplatin therapy [110]. Ardisianone, a natural benzoquinone, demonstrated a time-dependent a degradation of survivin and upregulation of cLAP1/2 expression in human refractory prostate cancer (HRPC) cell lines PC-3 and DU-145 following [128]. In a concentration-dependent manner, this molecule inhibited cell proliferation and induced both caspase-dependent and caspase-independent apoptosis via down-regulating Bcl-2 proteins, producing ROS, disrupting the mitochondrial membrane potential and interfering with the PI3K/Akt signaling pathway [128]. Given its ability to upregulate IAP expression, ardisianone might be a promising candidate for acquired chemotherapy- or IAP-antagonist resistance.

### Novel SMAC mimetic design

Whilst endogenous SMAC/DIABLO exerts its actions within micromolar ranges, SMAC mimetics such as AT-406 are more efficacious. This monomeric class of SMAC inhibitors exhibit strong binding affinities with XIAP and cIAP1/2 at nanomolar ranges [93]. To exert supraphysiological effects, researchers are continually working to improve the potency and apoptotic efficiency of novel SMAC mimetics to abolish drug resistance [129]. Reports of improved analogs resulted from the development of a second class of bivalent SMAC mimics that targeted more than one BIR domain region on XIAP, improving the rate of apoptosis [130]. A good example of a successful bivalent SMAC mimetic is ‘Birinapant’, currently undergoing phase 1 and 2 clinical trial for the treatment of ovarian cancer (Medivir, 2017) (outlined in Table 2).

Alternatively, researchers mutated the AVPI N-terminal sequence of SMAC which binds to the BIR domains of IAPs to IPVA (isoleucine–phenylalanine–valine–alanine) and FPVA (formic acid–phenylalanine–aline–alanine) using predictive computational analysis and induced fit docking models (Gold-score software). By doing so, the l-amino acids were substituted to d-amino acids, stabilizing the hydrophobic interaction of the SMAC mimetic within the IAP binding pocket whilst also preventing proteolytic action via XIAP BIR3 domains [131]. Results suggested that FPVA and AVPF were able to induce significantly improved apoptosis in L1236 cells and AVPF and AVPI sensitized etoposide resistance in L136 cells [131]. This computational approach provided an accurate predictive model for mutational analysis and as more information regarding SMAC mimetics becomes available, this can be further improved upon in future. Furthermore, the ability to dock inhibitors to the BIR3 domain of XIAP, instead of BIR2, better mimics the endogenous SMAC protein which has a higher affinity for BIR3 than BIR2 [131]. In this manner, apoptosis mediated by the binding between the retro-inverse SMAC peptide and the BIR3 domain of XIAP was greatly optimized. Favorably, these authors utilized Hodgkin Lymphoma cell lines L1236 and L428, with high endogenous expression of XIAP [131]. In going forward, it will be useful to assess the efficacy of retro-inverse SMAC peptides in alternative cancer cells types with varying levels of XIAP expression.

### Specificity of SMAC mimetics

As discussed, SMAC peptides that utilize XIAP as an IAP of interest whilst focusing on targeting the BIR3 domain of XIAP are most favorable as direct inhibitors of downstream caspases. XIAP inhibits both the extrinsic and intrinsic apoptotic pathways via direct inhibition of caspases, unlike cIAP1/2 which acts via proteasomal degradation or ubiquitination and may be limited by its initiation of cell protective effects via NF-kB signaling [132]. Critically, XIAP expression is up-regulated in a number of different types of cancers, some of which are intrinsically resistant to chemotherapy. Therefore, XIAP antagonism can be a powerful strategy to overcome chemo-resistance across a variety of cancers and a number of current IAP-antagonist strategies involve XIAP down-regulation or inhibition to promote cancer cell survival. Examples of compounds in development that target XIAP include SM-12d, Compound 21, AT-406 (Ascenta Therapeutics), LCL-161 (Novartis Pharmaceuticals), GDC-0152 (Genentech), GDC-0197 (Genentech), SM-164, Birinapant/ TL32711 (Tetralogic Pharmaceuticals), HGS1029, LBW-242, Compound 10 (Aegera Therapeutics), Compound 24 (Allist Pharmaceuticals) and Compound 1A (Genentech). Table 2 further outlines a list of prominent SMAC mimetics, their targets, cancer treatment profiles and progress in clinical trials.

It is important to note that whilst SMAC/DIABLO has better affinity for BIR3 and thus is a direct inhibitor of caspase 9 activation, the BIR2 domain is responsible for inhibiting terminal caspases 3 and 7 [133]. This research suggests that it could be advantageous to design novel, eqipotent mimetics that can target BIR2, thus facilitating modulation of both the intrinsic and extrinsic apoptotic pathways to amplify its effect. The specificity of various SMAC mimetics for BIR3 and its subsequent increased potency is outlined in detail in recent a patent review [134]. Some examples of patented potent molecules include the monovalent SMAC mimetic WO2014060767 (Astex Pharmaceuticals) which demonstrated 100% inhibition of cIAP1 BIR3 activity at concentrations as low as 12 nM, and 94% inhibition of XIAP BIR3 activity at 40 nM [134]. Interestingly, WO2014060767 is one of the few AVPI IAP antagonists without an alanine warhead in the SMAC peptide sequence. This approach is purported to create a more balanced binding affinity between XIAP and cIAP via a slight alteration in H-bond charge affinity that does not interfere too heavily with the conserved backbone of the molecule [135]. Other SMAC mimetic without an alanine warhead include WO2014060768 (Astex Pharmaceuticals) and WO2014060770 (Astex Pharmaceuticals), which are both potent SMAC inhibitors able to inhibit cIAP 1 and XIAP activity at low concentrations via binding at their respective BIR3 domains [134].

An example of a potent monovalent SMAC mimetic selective for cIAP1 BIR3 is Takeda’s JP2012176934 Compound 21. This small molecule inhibited 99% of cIAP1 activity at 3 µM whilst also inhibiting the proliferation of a MDA-MB-231 breast cancer cell line by 93% at concentrations as low as 0.1 µM [134]. Although claimed to exhibit substantial steric hindrance, some XIAP BIR2 selective monovalent SMAC mimetics also display extremely high potencies. Examples of these include Roche’s WO2014023708 Compound ‘1d’ and WO2014026882 which demonstrate IC50s as low as 0.029 and 0.013 µM, respectively [134]. Importantly, these molecules might be excellent candidates for combination therapy with anticancer agents, especially given that some are shown to reduce cell proliferation which can slow, or even halt tumor growth. However, their potential to overcome acquired and intrinsic chemotherapy resistance is so far unreported.

Some researchers suggest that the involvement of IAP regulation in bone metastasis and osteoclast differentiation might demonstrate significant clinical implications for patients undergoing IAP antagonist treatment [136]. They discussed the implications of targeting cIAPs and its actions on the alternative NF-kB signaling pathway that promote osteoclastogenesis via NIK stabilization and subsequent activation of differentiation inducing transcription factors such as NFATc1 [136]. These observations followed their study on the use of SMAC mimetic, BV6, in mouse model demonstrating both osteoporosis and increased tumor growth when 4T1 breast cancer cells were injected into the tibia [137]. Importantly, this tumorigenesis was limited to the bone-microenvironment and could be overcome by combining IAP antagonists with the antiresorptive drug, zoledronic acid [137]. Given that recent in vitro data reveals BV6 as a promising and effective single or combinatory therapeutic agent in re-sensitizing acute lymphoblastic leukemia (ALL) cells to chemotherapies [138], more information on the efficacy of SMAC mimetic in in vivo settings will be essential as this field progresses.

### SMAC mimetic resistance

Despite the fact that SMAC mimetics are designed to mimic their endogenous counterparts, albeit with improved potency, there are some cell types that exhibit intrinsic resistance to these synthetic compounds—often alongside resistance to chemotherapeutic agents.

An example of this phenomenon includes chronic lymphocytic leukemia (CLL) cells that are especially resistant to SMAC mimetics targeting cIAP1/cIAP2 activity [139]. It is understood that their resistance to SMAC mimetics, and possibly to chemotherapeutic agents, may be attributed to an elevation in aberrant NF-kB activity in ripoptosome-lacking CLL cells [139]. In cells lines sensitive to cIAP1/2 antagonizing SMAC mimetics, resistance to chemotherapy drugs can be overcome by TNF-α-mediated apoptosis following ripoptosome formation [140]. As the SMAC mimetic antagonizes, ubiquitylates and degrades cIAP1/2, a ripoptosome complex is formed via the assemblage of RIPK1 (receptor-interacting serine/threonine-protein kinase 1), FADD (Fas-associated protein with death domain), FLICE-like inhibitory protein and caspase-8, and can initiate autocatalysis and activation of caspase-8, which, in turn results in a sensitization to TNF-α-dependent apoptotic cell death [141]. However, resistant-CLL cells are unable to associate with one another to form a riptosome complex, despite degradation of cIAP1/2 by SMAC mimetic to induce TNF-α production and the presence of RIPK1, FADD, FLICE-like inhibitory protein and caspase-8 [139]. In this case, caspase-8 activation is reduced and the apoptosis-inducing caspase-cascade is minimized, rendering CLL cells resistant to SMAC mimetics.

Studies have reported that prolonged exposure (>3 h) of cancer cells to SMAC mimetic treatment results in increased expression levels of cIAP2 in CLL cells [139], lung carcinoma cells [140], colon carcinoma and melanoma carcinoma cells [142]. It was hypothesized that an increase in cIAP2 may be required for maintenance of resistance in these cell lines and therefore, this was more closely examined via PI3K inhibitor LY294002, known to suppress cIAP2 expression [139]. Whilst SMAC mimetic sensitivity was restored in lung carcinoma cells [140], it did not yield similar results in CLL cells [139]. These results suggest that targeting PI3K and NF-kB may be more useful in some cancer cell types than others, and that other factors modulating cIAP expression may be of relevance to the development and maintenance of SMAC mimetic resistance in CLL cells. Although it is clear that CLL cells do not respond to cIAP specific SMAC mimetics such as Compound A [139], the BIR3 specific SMAC mimetic (i.e. XIAP targeted) Smac066 improves sensitivity in CLL cells [143].

Of note, the implications of targeting cIAP1/2 mediated cell death in hematopoietic malignancies, such as CLL, remain controversial. Authors Lau and Pratt proposed that the effect of cIAPs on a cells physiological state is largely context-dependent, suggesting that whilst targeting cIAPs largely suppress tumorigenesis via their classical signaling action on caspases, the subsequent ubiquitination of cIAP can lead to constitutive NF-kB signaling, NIK ubiquitination, cell proliferation and tumor progression via a downregulation in pro-apoptotic p53 signaling [132]. Others suggest that cIAP targeting may have positive therapeutic synergy with cancer vaccines following a rapid sensitization to TNF-α signaling [144]. It will be important to consider targeting alternative pathways to address resistance to cIAP targeted SMAC mimetics in going forward. These might include further research into TNF-α mediated caspase-independent necroptosis, reported in apoptotic resistant cells lacking both FADD and caspase-8 expression that were stimulated and re-sensitized with the bivalent SMAC mimetic BV6 [145].

## Combinatory therapies to overcome resistance

### IAP antagonism and chemotherapy resistance

In recent years, in vitro studies have demonstrated that SMAC mimetics, in combination with anticancer drugs and TRAIL (TNF-related apoptosis-inducing ligand) can effectively enhance apoptosis and cell death in numerous cancer cell types, including T98G glioblastoma cells [146], HeLa cells [147] and lung adenocarcinomas [148]. Early research suggested that SMAC mimetics could enhance the sensitivity of anticancer agents’ paclitaxel, etoposide and doxorubicin in MCF-7 breast cancer cells [149]. Subsequently, researchers continued to investigate novel analogs of SMAC mimetics, revealing that SMAC mimetic also sensitized breast cancer cells to TRAIL-induced apoptosis treatment [150]. These results showed that growth was suppressed but apoptosis was not induced in some cell lines, such as T47D and MDA-MB-453, questioning their relevance to apoptosis-related cell death pathways [150].

Promising results have been shown for AT-406, an oral SMAC mimetic, in the sensitization of platinum based chemotherapy drugs such as carboplatin, cisplatin and paclitaxel in ovarian cancer cells [109]. Whilst AT-406 was an effective single agent for the treatment of OVCAR-8, SKOV-3, and OVCAR-3ip carboplatin-resistant ovarian cancer, the most promising results indicated sensitization to platinum resistant phenotypes in vitro and in vivo [109]. Induction of AT-406 mediated apoptosis in chemoresistance is understood to occur via downregulated expression of cellular XIAP, whereas cIAP1/2 degradation occurs in both sensitive and resistant cell lines [109]. XIAP dysregulation highlights AT-406 as an attractive SMAC mimetic, given that XIAP is an inhibitor of both intrinsic and extrinsic apoptotic pathways. Targeting both expression and inhibition via one compound truly maximizes its potential as an antagonist of IAPs. Refer to Table 2 for a clinical profile of AT-406.

Research to compare the effectiveness of SMAC mimetic analogs across differential cancer cell types are currently lacking. Bockbrader and colleagues demonstrated similar responses across multiple breast cancer cell types, and this is promising for broad spectrum breast cancer treatment [150]. However, future studies encompassing SMAC mimetic treatment across various cancer cell types will be essential. Within the last 5 years, potential therapeutic indications for small molecule inhibitor FL118 understood to act via inhibition of survivin, XIAP, cIAPs and Mcl-1 was reported [151]. This study demonstrated superior anti-tumorigenic activity in colon, lung, breast and prostate cancer cells exhibiting resistance to a range of first line chemotherapies. Moreover, these findings were replicated in a mouse model of head and neck cancer [151]. Researchers have now begun to investigate clinically compatible formulations of FL188, improving its toxicity and bioavailability [152].

In some instances, the discussed therapies may be ineffective as single-agents. When this occurs, combinatorial treatments should be explored. For example, the orally active SMAC mimetic LCL161 has been demonstrated to synergize with paclitaxel to restore chemosensitivity in hepatocellular carcinoma cells (HCCs) [110]. Other studies have demonstrated that the effectiveness of LCL161 was dependent on a low level of Bcl-2 protein expression in HCC cells [153]. However, despite relatively low Bcl-2 expression, hepatocellular carcinoma cell lines SNU423 and HuH7 were both intrinsically resistant to LCL161 [110]. This suggests that there may be more mechanisms contributing to SMAC resistance in hepatocellular carcinoma. This is currently unclear and research into alternative SMAC mimetic resistant cell types is needed. Even in combination with paclitaxel, apoptosis via LCL161 was induced with markedly low potency in SNU423 and HuH7 cells (>100 µM), [110]. Interestingly, as apoptosis increased, a decrease in cell proliferation was also reported, but XIAP and cIAP1/2 levels were unchanged. This might suggest that whilst combinatorial treatment enhanced the anti-proliferative effects in HCC cells, the pro-apoptotic effects may not solely be a result of IAP inhibition [110]. Of most concern, a recent study reported of a lymphoma mouse model that exhibited accelerated disease growth following treatment with LCL161, suggesting major contraindications for cLAP1/2 targeting via LCL161 in lymphoma [154]. In these instances, it is important to question the clinical relevance of SMAC mimetics targeting cIAPs for caspase-mediated apoptosis, given their potential to garner off-target effects via NF-kB signaling. Despite these concerns, several ongoing clinical trials are currently exploring LCL161 as a single and combinatory agent in the treatment of various cancers (Table 2).

### TRAIL resistance

TNF-related apoptosis-inducing ligand (TRAIL) binds to death receptors DR4 (TRAILRI) and DR5 (TRAILRII) on the cell membrane to induce apoptosis in cancer cells (refer to Fig. 1 for more detail on the cellular mechanism of TRAIL-induced apoptosis). When TRAIL associates with its receptor, there is a caspase-8 mediated cleavage of Bid, which becomes truncated-Bid (t-Bid) and promotes the activation of Bax [155]. This provides cross-talk between the extrinsic and intrinsic pathways, known as the mitochondrial amplification loop [15, 16]. Subsequently, mitochondrial PTPs release cytochrome c and the apoptosome is formed after aggregation of cytochrome c, pro-caspase-9, dATP and Apaf-1. This apoptotic caspase-cascade is initiated via the intrinsic apoptotic pathway [155].

Cells that respond to TRAIL signaling are categorized into two types; ‘type 2’ cells, such as hepatocytes and HCT-116 cells, require mitochondrial amplification (via intrinsic pathway) of the TRAIL death signal and ‘type 1’ cells, such as thymocytes, do not [156, 157]. Importantly, type 2 cells can become resistant to TRAIL therapy if anti-apoptotic members of the intrinsic apoptosis pathway, like Bcl-2, become dysregulated [158].

Cancer cells are reported to highly express TRAIL receptors, specifically DR4 and DR5, while healthy cells merely express ‘decoy’ receptors DcR1 and DcR2 [156]. Thus, TRAIL-induced apoptosis is an attractive way to combat cancer since it is highly specific for cancer cells and takes advantage of the patient’s immune cells, which also highly express these receptors [159]. Importantly, this minimizes cytotoxicity to normal, healthy tissue. Regardless of the immuno-modulatory function and specificity of TRAIL-therapy, many cancer cells are resistant to TRAIL following inadequate signal amplification via the intrinsic apoptosis pathway. A primary example of TRAIL resistance exists in ‘type 2’ colon cancer cells HCT-116 harboring a deficiency in Bax [160].

### IAP antagonism to overcome TRAIL resistance

The importance of XIAP antagonism in cancer treatment is additionally demonstrated by its ability to overcome TRAIL resistance. XIAP is responsible for the inhibition of effector caspases-3/7 via proximity induced proteasomal degradation at their active sites (Fig. 1) [161]. To sensitize ‘type 2’ cells to TRAIL therapy researchers have investigated the merits of repressing XIAP activity via SMAC mimetics [162], shRNA knockdown [162, 163] and Mithramycin A [164]—all with good success. Results demonstrate that SMAC mimetic, Sm-164 (a duel target of XIAP and cIAPs with a higher affinity for XIAP), was 1000-fold more potent in inducing apoptosis and restoring sensitivity to TRAIL than a cIAP specific SMAC mimetic [130]. Additionally, TRAIL-resistant prostate cancer cells expressed excessive amounts of XIAP and cIAP and exhibited improved TRAIL sensitivity when XIAP expression was knocked down [162]. Data supporting the involvement of XIAP in TRAIL resistance suggests that neutralizing XIAP might be critical for TRAIL sensitivity and an attractive target for potential combinatory therapy in the treatment of ‘type 2’ cancer cells. Additionally, XIAP also protects healthy ‘type 2’ cells from uncontrolled cell death. It has been suggested that broadly antagonizing XIAP as a sensitization strategy could lead to healthy hepatocyte death and subsequent liver damage [165].

### TRAIL receptors in TRAIL resistance

In addition to XIAP over-expression, insufficient expression of TRAIL receptors also contributes TRAIL resistance in many cancers, including in acute lymphoblastic leukemia cells (ALL). ALL cells demonstrate significantly altered cell-surface expression of DR4 in TRAIL resistance. Here, authors suggested that dysregulated receptor trafficking and increased receptor glycosylation may be of importance to TRAIL sensitivity [166]. Other conditions that hamper apoptosis include mutations in the death domain of TRAIL receptors or in the ligand binding pocket [167], and high expression of decoy antagonistic receptors [168]. The apoptotic potential of TRAIL has shown to improve via combinatory treatment with etoposide, doxorubicin or paclitaxel mediated upregulation of both DR4 (TRAILI) and DR5 (TRAILII) expression in numerous breast cancer cells types in vitro, as well as in tumorigenic mice [169]. The anti-tumor antibiotic Bleomycin and the histone deacetylase inhibitor MS-275 have also upregulate DR4 and DR5 to sensitize cancer cells to TRAIL-induced apoptosis [170, 171]. Lastly, microtubulin targeting compound PBOX-15 (pyrrolo-1,5-benzoxazepine) treatment in myeloma in Jurkat ALL cells also resulted in an upregulation of DR5 to enhance TRAIL-induced apoptosis [172].

### Bcl-2 expression in TRAIL resistance

Relating specifically to activation of the intrinsic apoptotic pathway, HCT-116 wild type cells demonstrated a sensitization to apoptosis when Mcl-1 was inhibited. Mcl-1 selectively inhibits Bax/Bak signaling, preventing cross-talk between the death receptor pathway and the intrinsic apoptotic pathway [26]. Improved sensitivity to TRAIL was reported following release of endogenous SMAC when Mcl-1 was found to be overexpressed [173]. This might highlight further therapeutic implications for ‘pan’ inhibitor FL118 targeting both XIAP and Mcl-1, alongside survivin and cIAP1/2 [151]. Interestingly, it was recently shown that Bax activation is Mcl-1 independent in some cell types (including HCT-116 cells), rendering its inhibition an ineffective single agent treatment [174].

Sensitivity to TRAIL resistance is further demonstrated by upregulated expression of pro-apoptotic agents as Bax/Bak. In 2011, increased Bax expression and subsequent improved sensitivity to TRAIL was reported in colon cancer cells following exposure to the plant derived compound Nimbolide [175], although it is important to note that this article has since been retracted by the publisher. Other research suggests that Nimbolide exerts its anti-tumorigenic effects via a downregulation in cell proliferation and metastasis [176]. Whilst these strategies promote apoptotic cell death, they do not appear to be effective in switching ‘type 2’ cells to mitochondrial pathway independent ‘type 1’ cells.

Other than XIAP repression, Mcl-1 inhibition and proteasome inhibition, resistance to TRAIL may be overcome by either kinase inhibitors or BH3 mimetics. Kinase inhibitors such as Roscovitine and Sorafenib (approved for HCC treatment) suppress activation of Mcl-1 [177], downregulate expression of c-FLIP [178], and aid in DISC-pro-caspase-8 activation to facilitate apoptosis [179]. In a similar fashion, BH3 mimetic ABT-737 represses pro-survival Bcl-2 proteins, such as Mcl-1, to further exert a pro-apoptotic effect via Bax/Bak signaling in chemoresistance [180]. However, ABT-737 is Bax/Bak dependent and would not be effective in double deficient HCT-116 cells, or Mcl-1 independent cells [174].

For Bax-/Bax-deficient HCT-116 cells, proteasome inhibitors MG132 and Bortezomib (BZM) sensitized TRAIL resistance in HCT-116 Bax-/Bak-double deficient cells [181]. Some TRAIL-resistant cancer cells are neither Bax/Bak deficient, nor do they have abnormally low expression of DR4 and DR5. Herein, protein analysis revealed high levels of c-FLIP, anti-apoptotic Bcl-2 members and IAPs [182]. It is likely that c-FLIP (cellular FLICE inhibitory protein), Mcl-1 and IAPs such as XIAP and cIAP all contribute to TRAIL resistance. c-FLIP tends to promote TRAIL-resistance in malignant cancer cells by preventing the formation of DISC [183]. TRAIL resistance was improved via siRNA knockdown or drug induced downregulation of c-FLIP via rocaglamide combined with SMAC mimetic AT406 [182]. Enhanced TRAIL-induced apoptosis was previously demonstrated using the flavonoid kurarinone by down-regulating cFLIP in HeLa cells [184].

### IAPs and antibody-based therapy resistance

Drozitumab is a human monoclonal antibody that binds specifically to TRAIL receptor DR5 on cancer cells to induce apoptosis [185]. Most breast cancer cells, including the basal-like MDA-MB-231-TXSA cells, are extremely sensitive to drozitumab-induced apoptosis, but prolonged exposure can induce acquired resistance to the cytotoxic agent [186]. There is no correlation between sensitivity to Drozitumab and expression of DR4/5 in cancer cells, unlike in TRAIL resistant cells. However, like TRAIL resistant cells, these resistance-causing factors may, in part, be mediated by IAPs. Demonstrating this, the pan IAP antagonist SMAC mimetic BV6 used in combination with Drozitumab restored its sensitivity and completely inhibited the growth of MDA-MB-TXSA tumors in a mouse model [112]. Interestingly, this drug combination was independent of TNF-α and was successful in the direct activation of effector caspase-3 and -7, suggestive of XIAP antagonism [112]. Moreover, this combination bypassed activation of the mitochondrial amplification loop, commonly required in overcoming TRAIL receptor related resistance [112].

The chemotherapeutic agent doxorubicin has also been reported as an effective sensitizer to Drozitumab via suppressive IAP expression, as well as increased cell-surface expression of DR5 [186]. This also improved sensitivity in vivo whereby inhibited tumor growth, delayed tumor progression and improved chances of survival were reported in mice treated with both doxorubicin and Drozitumab [186]. Given that part of its improved sensitivity was via differential expression of DR5, it could be suggested that this combination might exhibit less toxicity in healthy cells that do not normally express this receptor. Interestingly, Drozitumab-resistant cells also demonstrated sensitivity to taxol, etoposide, cisplatin, and the deacetylase inhibitor, SAHA [186]. Unsurprisingly, patients with acquired resistance to TRAIL are also reportedly cross-resistant to drozitumab-induced apoptosis and the authors suggest potential benefits for patients with drozitumab resistance that switch to TRAIL therapy or explore combination therapies [186].

## Conclusion

Our dependency upon a core group of chemotherapeutics as first line treatment is continually reflected by the generation of acquired chemoresistance across all cancer types. The ever-growing burden of resistance highlights the requirement for a more diverse set of therapeutics in cancer treatment. Though the development of resistance to anticancer agents involves multiple differential pathways, often dependent on drug and tissue type, they share commonality in an overall reduction in cancer cell death. Apoptosis is the primary mediator of chemotherapy mediated cell death and its regulation is not unaffected in the development of resistance. In this review, we have discussed the recent advances in research toward targeting apoptotic pathways in cancer treatment and resistance, with a central focus on the modulation of IAPs. Whilst various combinatorial therapeutic approaches for use of antagonists of IAPs following the development of resistance to chemotherapy, antibody-based therapy and TRAIL resistance were discussed [150, 151, 185], we also focused upon resistance to IAP antagonism itself which may have future implications in the clinic [139, 141].

Research characterizing the expression and regulation of IAPs in disease has facilitated the identification of novel therapeutic options within this field, particularly in relation to the expression and inhibition of XIAP and cIAP1 and 2 [14]. The use of SMAC mimetics as means to exploit IAP overexpression has advantages for specific tumor types [187].

Although original research and clinical studies suggest that IAPs may be effective as single agents in cancer, ongoing clinical studies mostly assess the usefulness of IAP antagonists in combination with alternative cancer treatments to re-sensitize chemotherapy in relapsed cancers [188]. As research progresses, improvements in their therapeutic design may enhance their affinity and specificity for IAP mediated cell death.

This review also highlights the implications for treatment in relation to the development of acquired resistance to IAP antagonists and, indeed, intrinsic resistance that is demonstrated in CLL cell types [139, 143]. These potential limitations warrant scientific discussion to devise strategies for overcoming resistance to IAP antagonism before they become an issue in the clinic. In regards to sensitizing cells to IAP-antagonists, or augmenting the cytotoxic activity of other agents, a wealth of scientific data is available to suggest combinatorial treatments offer the most practical solution.

## Abbreviations

AIF: Apoptosis inducing factor
ALL: Acute lymphoblastic leukemia
AML: Acute myeloid leukemia
ANT: Adenine nucleotide translocator
Apaf1: Apoptosis protease activating factor 1
API2: Apoptotic protein inhibitor 2
Asp: Aspartate
AVPF: Alanine–valine–phenylalanine–formic acid
AVPI: Alanine–valine–phenylalanine–isoleucine
Bad: Bcl-2 associated death promoter
Bak: Bcl-2 antagonist and killer
Bax: Bcl-2 associated X protein
Bcl-2 proteins: B cell lymphoma 2 proteins
Bcl-Xl: B cell lymphoma-extra large
Bid: BH3 interacting-domain death agonist
Bim: Bcl-2-like protein 11
BIR domain: Baculovirus IAP repeat domains
BIRC: Baculoviral inhibitors of apoptosis repeat containing proteins
Bmf: Bcl-2 modifying factor
BRUCE: BIR-containing ubiquitin conjugating enzyme
BZM: Bortezomib
CAD: Caspase activated DNAase
CARD: Caspase-recruitment domain
CDDP: Cisplatin
CD95: Cluster of differentiation 95
Chk1: Checkpoint kinase 1
cIAP1: Cellular inhibitor of apoptosis protein 1
cIAP2: Cellular inhibitor of apoptosis protein 2
CK: Creatine kinase
CLL: Chronic lymphocytic leukemia
CML: Chronic myelogenous leukemia
CypD: Cyclophilin D
Cys: Cysteine
Cyt c: Cytochrome c
dATP: Deoxyadenosine triphosphate
DISC: Death-inducing signaling complex
DR4: Death receptor 4
DR5: Death receptor 5
FADD: Fas-associated protein with death domain
FasL/R: Fas ligand/receptor
FLASH: Flice-associated huge protein
FPVA: Formic acid–phenylalanine–valine–alanine
HCC: Hepatocellular carcinoma cells
HER2: Human epidermal growth factor receptor 2
HK: Hexokinase
HIAP2: Human inhibitor of apoptosis 2
hILP1/2: Human IAP-like protein 1/2
HRPC: Human refractory prostate cancer
IAPs: Inhibitors of apoptosis proteins
IBM: IAP binding motifs
ILP-1/2: IAP-like protein 1/2
IMM: Inner mitochondrial membrane
IPVA: Isoleucine–phenylalanine–valine–alanine
IV: Intravenous
KIAP: Kidney inhibitor of apoptosis protein
LRR domain: Leucine-rich repeat domain
MALT Lymphoma: Mucosa-associated lymphoid tissue lymphoma
MAP/Akt proteins: Microtubule associated proteins/protein kinase B
Mcl-1: Myeloid cell leukemia 1
MDS: Myelodysplastic syndromes
MIHA/B/C: Mammalian homolog of IAP A/B/C
ML-IAP: Melanoma inhibitor of apoptosis
MOMP: Mitochondrial outer membrane permeabilization
NACHT domain: NAIP, C2TA, HET-E and TP1 domain
NAIP: Neuronal apoptosis inhibitory protein
NFkB pathway: Nuclear factor kappa beta pathway
Omi/HtrA2: Temperature requirement protein A2
OMM: High outer mitochondrial membrane
PBR: Peripheral benzodiazepine receptor
PBOX-15: Pyrrolo-1,5-benzoxazepine
PM: Plasma membrane
PTPs: Permeability transition pores (mitochondrial)
RING domain: Really interesting new gene domain
RIPK1: Receptor-interacting serine/threonine-protein kinase 1
RNAi: Ribonucleic acid interference
ROS: Reactive oxygen species
SADS: Small accelerator of death signaling
SAHA: Suberoylanilide hydroxamic acid
siRNA: Small interfering RNA
SMAC/DIABLO: Second mitochondrial derived activator of caspase/direct inhibitor of apoptosis-binding with a low isoelectric point
tBid: Truncated-bid
TNF: Tumor necrosis factor
TNF-α: Tumor necrosis factor alpha
TNFαR1: TNF-α receptor 1
TNFR: TNF-α receptor complex
TP53: Tumor protein 53
TRADD: TNFRSF1A-associated via death domain
TRAF2/5: TNF-receptor associated factor 2/5
TRAIL: TNF-related apoptosis-inducing ligand
TRAILRI/II: TRAIL receptor I and II
Ts-IAP: Testis-specific inhibitor of apoptosis protein
VDAC2: Voltage-dependent anion channel 2
XAF1: XIAP-associated factor 1
XIAP: X-linked inhibitor of apoptosis protein
XLP: X-linked proliferative disorder
